# Novel HER2-Directed Treatments in Advanced Gastric Carcinoma: AnotHER Paradigm Shift?

**DOI:** 10.3390/cancers13071664

**Published:** 2021-04-01

**Authors:** Angela Dalia Ricci, Alessandro Rizzo, Fabiola Lorena Rojas Llimpe, Francesca Di Fabio, Dario De Biase, Karim Rihawi

**Affiliations:** 1 Division of Oncology, IRCCS Azienda Ospedaliero—Universitaria di Bologna, 40138 Bologna, Italy; angeladalia.ricci@studio.unibo.it (A.D.R.); fabiolalorena.rojas@aosp.bo.it (F.L.R.L.); francesca.difabio@aosp.bo.it (F.D.F.); karim.rihawi@aosp.bo.it (K.R.); 2 Department of Experimental, Diagnostic & Specialty Medicine, Azienda Ospedaliero—Universitaria di Bologna, Via Massarenti 13, 40138 Bologna, Italy; 3 Department of Pharmacy and Biotechnology (FABIT), University of Bologna, 40138 Bologna, Italy; dario.debiase@unibo.it

**Keywords:** HER2, ErbB2, gastric cancer, trastuzumab deruxtecan, trastuzumab

## Abstract

**Simple Summary:**

More than ten years after the first publication of the results of the ToGA phase III trial, active research avenues are exploring the role of novel agents targeting human epidermal growth factor receptor 2 (*HER2*). Preliminary reports have highlighted promising activity for several therapeutics, including the recently approved trastuzumab deruxtecan in pretreated GC patients. Herein, we discuss novel therapeutic opportunities in this setting.

**Abstract:**

Human epidermal growth factor receptor 2 (*HER2*) is overexpressed and/or amplified in approximately 15–20% of gastric adenocarcinoma (GC) patients. In 2010, the landmark ToGA trial established the combination of trastuzumab plus chemotherapy as the first-line standard of care for *HER2*-positive GC patients with advanced disease. However, subsequent studies on HER2 targeted therapies in this setting failed to meet their primary endpoints, and not all *HER2*-positive GC patients benefit from targeted approaches. More recently, novel *HER2*-directed treatments have been investigated, including trastuzumab deruxtecan (T-Dxd); following the results of the DESTINY-Gastric01 study, T-Dxd received its first U.S. Food and Drug Administration (FDA) approval on 15 January 2021 for the treatment of adults with unresectable, locally advanced, or metastatic GC who have received a prior trastuzumab-based regimen. In this review, we discuss the current *HER2*-targeted treatments for GC in the advanced disease setting, mainly focusing on emerging new treatments and future research directions.

## 1. Introduction

Gastric adenocarcinoma (GC) remains a global health problem, representing the fifth most commonly diagnosed malignancy, the third leading cause of cancer-related death worldwide, and accounting for more than one million cases annually [1]. Unfortunately, many GC patients experience relapse following radical surgery or are diagnosed with advanced disease, where systemic treatment represents the only therapeutic option with palliative intent [2,3].

The last decade has registered the emerging of novel therapeutic targets in advanced GC, including Programmed Cell Death 1 (PD-1) and *HER2*, and the subsequent introduction of immunotherapy and *HER2* inhibitors in clinical practice following the results of landmark clinical trials in this setting [4,5,6]. *ERBB2* is the gene encoding HER2, with the latter that is commonly dysregulated in GC, as shown by several studies consistently showing that *HER2* mutations, fusions, amplifications, and other aberrations are a frequent finding in gastric and gastroesophageal (GEJ) cancers [7]. In addition, it is widely recognized that *HER2* expression varies according to the anatomical site of GC (fundus, pylorus, antrum, etc.) and to the GC molecular subtype identified by The Cancer Genome Atlas (TCGA) project [8]. Moreover, GCs and GEJ cancers with *HER2* amplification are more commonly moderately- or well-differentiated intestinal forms [9]. Overall, a proportion between 15% and 20% of all GCs is deemed to express *HER2*; however, a key element to consider when discussing *HER2* assessment is the high degree of intratumoral heterogeneity in GC compared to other solid tumors such as breast cancer [10]. As a matter of fact, staining patterns may be different according to the tumor site and the type of specimen tested (surgical versus biopsy specimen, especially due to the reliability of staining patterns in small biopsies compared to large tumor specimens) [11]; thus, specific guidelines have been formulated for *HER2* assessment in GC, with *HER2*-positive disease defined by different criteria on the basis of the staining pattern of the surgical specimen (strong complete, basolateral, or lateral membranous reactivity in ≥10% of tumor cells) or the biopsy specimen (tumor cell cluster with strong complete, basolateral, or lateral membranous reactivity irrespective of % of tumor cells stained) (Table 1) [12].

In the last decade we have witnessed the development of an impressive number of *HER2* targeted therapies, with many of them being investigated in GC patients [13,14,15]. However, since the publication of the pivotal ToGA trial which established the combination of fluoropyrimidine-cisplatin plus trastuzumab as the standard of care in treatment-naïve patients with *HER2*-positive metastatic GC, a plethora of negative trials on *HER2* targeted therapies in GC patients have been presented or published [16]; in fact, the LOGiC, TyTAN, JACOB, and GATSBY trials on lapatinib, pertuzumab, and trastuzumab emtansine (T-DM1) failed to meet their primary endpoints [17,18,19,20] (Table 2). More recently, we have seen the development of novel and promising *HER2* targeted treatments as monotherapy or in combination with other anticancer agents, some of which have already reported interesting results in early-phase clinical trials [21].

In this review, we present an overview regarding the state of the art of HER2-directed treatments in advanced GC, especially focusing on recently published studies and ongoing clinical trials on novel *HER2* targeted treatments. We performed a research on Pubmed/Medline, Cochrane Library, and Scopus using the keywords “gastric cancer” OR “gastric adenocarcinoma” OR “gastroesophageal cancer” AND “*HER2*” OR “human epidermal growth factor receptor” OR “trastuzumab” OR “trastuzumab deruxtecan” OR “margetuximab” OR “ZW25” OR “trastuzumab emtansine”. We selected pivotal registration studies. We also selected the most relevant and pertinent studies considering the quality of the studies in terms of their applicability, how they were conducted, statistical analysis, number of patients enrolled, and outcomes. For ongoing clinical trials, we searched in the clinicaltrials.gov database for recruiting and active, not recruiting trials, using the following keywords: “gastric cancer” OR “gastric adenocarcinoma” OR “gastroesophageal cancer” AND “*HER2*” OR “human epidermal growth factor receptor” OR “trastuzumab” OR “trastuzumab deruxtecan” OR “margetuximab” OR “ZW25” OR “trastuzumab emtansine”. We restricted our research to phase I, II, or III trials focused on the metastatic/advanced setting.

## 2. Trastuzumab, the ToGA Trial and Resistance Mechanisms

More than ten years ago, the publication of the landmark ToGA trial ushered in a new era in GC management, leading to the approval of the combination of chemotherapy plus trastuzumab in the front-line setting of *HER2*-positive metastatic GC [16]. In this phase III, randomized, open-label, multicenter study, 584 treatment-naïve HER2-positive GC patients with advanced disease were randomly allocated to fluoropyrimidine (5-fluorouracil or capecitabine at investigator’s discretion) plus cisplatin or to fluoropyrimidine-cisplatin plus trastuzumab [16]; the results of the study showed a statistically significant OS benefit in *HER2*-positive patients assigned to trastuzumab plus chemotherapy compared with chemotherapy alone, with median OS of 13.8 months and 11.1 months, respectively (Hazard Ratio [HR], 0.74; Confidence Interval [CI], 0.60–0.91; *p* = 0.0046) [16]. In addition, the ToGA suggested that some subgroups of GC patients could derive greater advantage, with the extent of the benefit related to the degree of *HER2* expression or amplification [16]. In fact, an exploratory post-hoc analysis focused on immunohistochemistry (IHC)2+/fluorescent in situ hybridization (FISH)-positive or IHC3 + GCs, where the combination therapy with trastuzumab reported a median OS of 16 months versus 11.8 months in the chemotherapy group (HR, 0.68; 95% CI, 0.5–0.83) [24].

Unfortunately, trastuzumab resistance represents a major obstacle in *HER2*-positive patients as it inevitably occurs [25]. Despite some mechanisms being shared between breast cancer and GC (e.g., the activation of downstream pathway, low levels of *HER2* expression, etc.), several studies have suggested that there are other mechanisms of resistance that are specifically involved in GC [26]. Among these, a number of reports have highlighted that *HER2* expression may be lost in GC patients following disease progression on trastuzumab [27,28]. In addition, unlike breast cancer, several aberrations are able to drive trastuzumab resistance in GC, including mutations in *EGFR*, *MET*, *KRAS*, *PI3K*, and *PTEN* genes, as well as *EGFR*, *MET*, and *KRAS* amplifications [29].

The development and introduction of immune checkpoint inhibitors (ICIs) in GC has spurred a growing interest in combination strategies including ICIs and *HER2*-targeted treatments [30]. In fact, preclinical studies have suggested a synergistic activity for the combinations including trastuzumab and PD-1 inhibitors, resulting in T-cell activation and enhancing Antibody Dependent Cellular Phagocytosis (ADCC) in murine models [31]. In a recent phase II trial, *HER2*-positive treatment-naïve patients receiving pembrolizumab, trastuzumab, platinum and fluoropyrimidine reported interesting results, with a 91% of response rate and median overall survival (OS) of 27.3 months [32]. Ongoing clinical trials will probably shed further light on the role of this combination strategy, with the highly awaited results of a phase III study of trastuzumab-chemotherapy plus pembrolizumab versus trastuzumab-chemotherapy plus placebo in the front-line setting (NCT03615326).

## 3. Trastuzumab Deruxtecan

**Figure 1:**
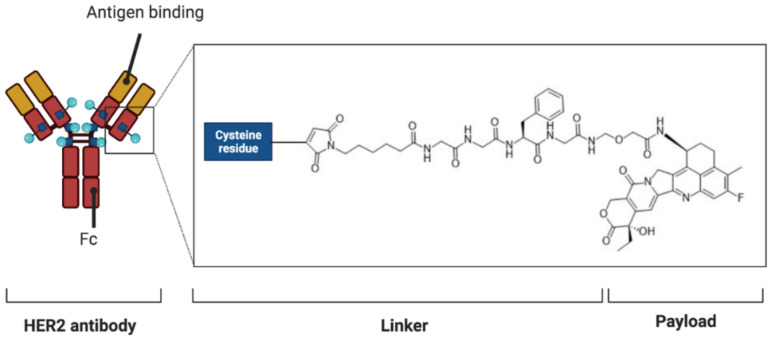
Schematic figure representing trastuzumab deruxtecan (T-Dxd). T-Dxd is composed of an anti-*HER2* antibody, a cleavable tetrapeptide-based linker, and deruxtecan—a topoisomerase I inhibitor. The maleimide group in deruxtecan is delivered by the antibody.

Trastuzumab deruxtecan (T-Dxd, or DS-8201a) is a second-generation antibody-drug conjugate (ADC) with a DNA topoisomerase I inhibitor [33]. More specifically, T-Dxd presents three different parts: the *HER2* antibody, a tetrapeptide-based linker, and a topoisomerase I inhibitor payload (Figure 1) [34]. Preclinical data showed that T-Dxd is also effective in *HER2*-low tumors, as opposed to other *HER2* inhibitors [35].

Firstly, a phase I study conducted by Shitara and colleagues reported promising levels of activity in 44 *HER2*-positive GC patients with advanced disease receiving T-Dxd at a dose of 5.4 or 6.4 mg/kg (NCT02564900) [36]. In particular, the trial included highly pretreated patients, with a median of three prior chemotherapy regimens; in this study, the overall response rate (ORR) was 43.2%, with a median duration of response of 7 months, and median progression-free survival (PFS) and overall survival (OS) of 5.6 months and 12.8 months, respectively.

More recently, the results of the DESTINY-Gastric01 study have been presented and published (NCT03329690) [23]. In this open-label, multicenter, randomized phase II trial, 187 *HER2*-positive GC patients who had previously received at least two lines of treatment, were enrolled in Korea and Japan [23]. Patients were randomly allocated to T-Dxd (*n* = 125) or to physician’s choice (*n* = 62), consisting of paclitaxel or irinotecan monotherapy [23]. The primary analysis of the DESTINY-Gastric01 included IHC 3+ or IHC 2+/ISH+ GC patients whose disease progressed on a trastuzumab-containing regimen [23]. The ORR, primary endpoint of the trial, was 51.3% (95% CI, 41.9–60.5) and 14.3% (95% CI, 6.4–26.2) in the T-Dxd and the physician’s choice group, respectively, with a confirmed disease control rate (DCR) of 85.7% in the experimental arm. In addition, the median confirmed duration of response (DOR) was 11.3 months in patients treated with T-Dxd compared to 3.9 months in the physician’s choice arm, and ten (8%) patients of the T-Dxd group achieved complete response—versus none in the control arm [23]. OS and PFS were the secondary endpoints of the DESTINY-Gastric01; T-Dxd reported an improvement in terms of median OS of 4.1 months, with median OS of 12.5 and 8.4 months in the T-Dxd and the physician’s choice groups, respectively (HR, 0.59; CI, 0.39–0.88; *p* = 0.0097) [23]. Similarly, the median PFS in the two groups was 5.6 months and 3.5 months, favoring the experimental treatment (HR, 0.47; CI, 0.31–0.71). Although the DESTINY-Gastric01 has represented an important step forward, the results of this phase II study have raised some controversies in the GC medical community. In fact, the magnitude of benefit seemed pronounced in the IHC 3+ population while the ORR, PFS, and OS benefit was not confirmed in IHC 2+ or ISH-positive GC patients, where a lower response rate was observed [23,37]. In addition, the study presented limited ethnic diversity, with all patients enrolled in Asian countries; lastly, the safety profile of T-Dxd needs to be considered, since the 10% of patients of the experimental arm experienced interstitial lung disease, although most were defined as grade II. As such, a careful risk-benefit assessment should be performed in presence of underlying lung injury [23,37].

On 15 January 2021, the United States (U.S.) Food and Drug Administration (FDA) approved T-Dxd for adult patients with metastatic GC who have received a prior trastuzumab-based regimen, with the agent representing the second *HER2* targeted treatment approved for *HER2*-positive GC. T-Dxd had previously been approved in Japan on 25 September 2020 with the same indications. Moreover, the efficacy and safety of T-Dxd is also under evaluation in an ongoing phase II, open-label, single-arm trial in Western countries, which is currently enrolling previously treated patients with advanced GC or GEJ cancers (NCT04014075). In contrast to the DESTINY-Gastric01, the study requires the central confirmation of the *HER2* status on new tissue sample; lastly, the trial has ORR as the primary endpoint, based on an independent central review, and PFS and OS as secondary endpoints.

## 4. Margetuximab

Margetuximab is an Fc (fragment crystallizable region) engineered *HER2*-directed monoclonal antibody with enhanced ADCC activity [38]; notably enough, promising antitumor activity has been reported in early-phase clinical trials on HER2-positive cancer patients, including low *HER2*-expressing GC [39]. In particular, the combination of the PD-1 inhibitor pembrolizumab plus margetuximab has shown promising levels of activity in a phase Ib-II trial in *HER2*-positive GCs and GEJ cancers, with an ORR of 35.7% and a DCR of 67.9% in the *HER2* 3+ and PD-L1 positive patient population [40]. Based on this biological rationale and this preliminary evidence, the combination of the PD-1 inhibitor plus margetuximab seems to show a synergistic activity, with a favorable safety profile. This preliminary evidence has supported further research in the same direction, as witnessed by the ongoing MAHOGANY randomized, open-label phase II/III trial, comprising different cohorts and parts. In fact, the first part of cohort A MAHOGANY aims at assessing the ORR and the tolerability of the combination therapy of margetuximab plus the PD-1 inhibitor retifanlimab (MGA012) in *HER2*-positive (IHC 3+ or IHC 2+/FISH-positive) GCs [39]; conversely, in cohort B patients with *HER2*-positive GC, regardless of the PD-L1 status, are randomized to receive (1) combination of chemotherapy plus trastuzumab, (2) chemotherapy plus margetuximab, (3) chemotherapy plus margetuximab plus retifanlimab, or (4) the PD-1 and LAG-3 inhibitor tebotelimab [39].

## 5. ZW25, Tucatinib, and Other HER2-Targeted Agents

**Figure 2:**
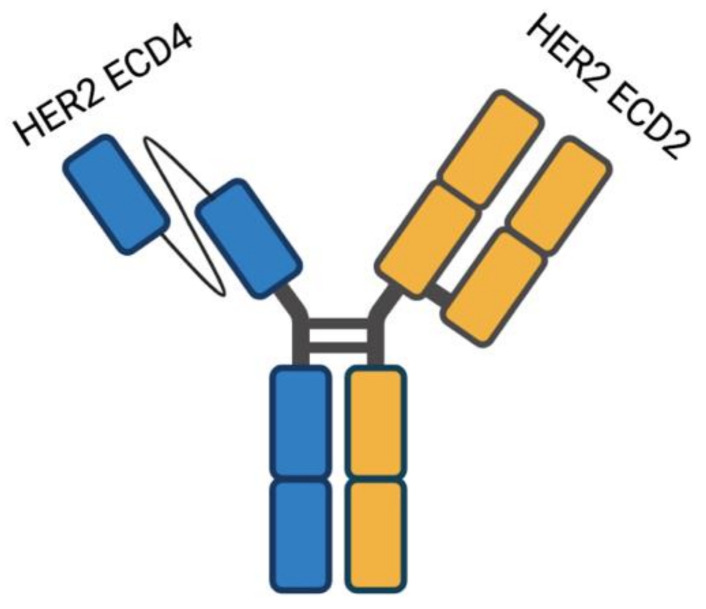
Schematic structure of ZW25, presenting a unique binding geometry and mechanism of action. ZW25 is a bispecific *HER2*-directed antibody able to bind two *HER2* epitopes: the pertuzumab binding domain ECD2 and the trastuzumab binding domain ECD4.

Another molecule under evaluation in this setting is the *HER2*-targeted bispecific antibody ZW25 which binds to two distinct *HER2* epitopes (Figure 2) [41]. In preclinical studies, ZW25 showed promising activity in HER2-positive cancer cells lines and in breast, gastric, and ovarian CDX (Cell line-Derived Xenograft) and PDX (Patient-Derived Xenografts) models [42]. In a phase I study evaluating ZW25 in *HER2*-positive solid tumors, four out of nine GC/GEJ cancer patients achieved partial response (PR) [43]; in addition, ZW25 is being evaluated in combination with different chemotherapeutic agents in treatment-naïve *HER2*-positive patients.

Besides the previously discussed agents, there are also some tyrosine kinase inhibitors (TKIs) under assessment in *HER2*-positive disease. Among these, the second-generation TKI tucatinib (ONT-380) has been suggested to be more potent than lapatinib with also a more favorable safety profile [44]. Previous clinical trials evaluating tucatinib in breast cancer have reported impressive results, especially in pretreated patients with brain metastases [45]; moreover, this molecule has the potential to represent a promising therapeutic option when combined with other anticancer agents, given the encouraging responses observed in other malignancies—Including colorectal cancer [46]. To date, few data are available in GC, where some clinical studies are evaluating this agent (NCT04430738, NCT04499924).

Among the number of *HER2*-directed agents, there is a growing attention towards MT-5111, an immunotoxin whose action is based on the use of a Shiga-like toxin to enter into *HER2*-positive cells [47]; notably enough, preclinical reports have suggested that MT-5111 could be combined safely with trastuzumab, and MT-5111 is being investigated in breast cancer and GC patients [48].

## 6. Conclusions

For more than ten years trastuzumab has represented the only approved targeted treatment for *HER2*-positive GC, with several phase II and III clinical trials evaluating other *HER2*-targeted agents such as lapatinib, T-DM1, and pertuzumab failing to meet their primary endpoints. However, T-Dxd has been recently approved by the US FDA, following the results of the DESTINY-Gastric01 trial, and adding to available treatment options in this setting. Several novel therapies are under investigation, including margetuximab, ZW25 and combination strategies including chemotherapy, *HER2*-targeted therapies, and PD-1.

## Figures and Tables

**Table 1:** Human epidermal growth factor receptor 2 (HER2) positivity in gastric cancer—according to immunohistochemistry and different specimens (surgical versus biopsy staining pattern).

Score	Surgical Specimen Staining Pattern	Biopsy Specimen Staining Pattern	*HER2*
0	No reactivity or membranous reactivity in <10% of tumor cells	No reactivity or no membranous reactivity in any tumor cell	Negative
1+	Faint or barely perceptible membranous reactivity in ≥10% of tumor cells; cells are reactive only in part of their membrane	Tumor cell cluster with faint or barely perceptible membranous reactivity irrespective of % of tumor cells stained	Negative
2+	Weak to moderate complete, basolateral or lateral membranous reactivity in ≥10% of tumor cells	Tumor cell cluster with weak to moderate complete, basolateral or lateral membranous reactivity irrespective of % of tumor cells stained	Equivocal (need ISH testing)
3+	Strong complete, basolateral or lateral membranous reactivity in ≥10% of tumor cells	Tumor cell cluster with strong complete, basolateral or lateral membranous reactivity irrespective of % of tumor cells stained	Positive

Abbreviations: ISH: In Situ Hybridization.

**Table 2:** Phase II and III randomized clinical trials evaluating *HER2*-directed treatments in gastric cancer.

Study [Ref]	Study Design	Treatment Arm (A)	Treatment Arm (B)	Setting
ToGA [16]	Open-label, international, phase III, randomized	Trastuzumab + CDDP + 5FU/Cape	CDDP + 5FU/Cape	First-line
LOGiC [17]	Double-blind, international, phase III, randomized	Lapatinib + CapeOX	Placebo + CapeOX	First-line
TyTAN [18]	Two-part, Asian, parallel-group, phase III	Lapatinib + paclitaxel	Paclitaxel	Second-line
GATSBY [19]	Open-label, international, phase II/III, randomized	T-DM1	Taxane	Second-line
JACOB [20]	Double-blind, international, phase III, randomized	Pertuzumab + trastuzumab + CDDP + 5FU/Cape	Placebo + trastuzumab + CDDP + 5FU/Cape	First-line
WJOG7112G (T-ACT Study) [22]	Open-label, Japanese, phase II, randomized	Trastuzumab + paclitaxel	Paclitaxel	Second-line
DESTINY-Gastric01 [23]	Open-label, Asian, phase II, randomized	Trastuzumab deruxtecan	Paclitaxel or Irinotecan	Third-line and later-lines

Abbreviations: 5FU: 5-fluorouracil; Cape: capecitabine; CapeOX: capecitabine plus oxaliplatin; CDDP: cisplatin; ref, reference; T-DM1: trastuzumab emtansine.

## Data Availability

Not applicable.

## References

[1] Bray F., Ferlay J., Soerjomataram I., Siegel R.L., Torre L.A., Jemal A. (2018). Global cancer statistics 2018: GLOBOCAN estimates of incidence and mortality worldwide for 36 cancers in 185 countries. CA Cancer J. Clinic..

[2] Ricci A.D., Rizzo A., Brandi G. (2021). DNA damage response alterations in gastric cancer: Knocking down a new wall. Futur. Oncol..

[3] Nagaraja A.K., Kikuchi O., Bass A.J. (2019). Genomics and targeted therapies in gastroesophageal adenocarcinoma. Cancer Discov..

[4] Vrána D., Matzenauer M., Neoral Č., Aujeský R., Vrba R., Melichar B., Rušarová N., Bartoušková M., Jankowski J. (2018). From tumor immunology to immunotherapy in gastric and esophageal cancer. Int. J. Mol. Sci..

[5] Rizzo A., Mollica V., Ricci A.D., Maggio I., Massucci M., Limpe F.L.R., Di Fabio F., Ardizzoni A. (2020). Third- and later-line treatment in advanced or metastatic gastric cancer: A systematic review and meta-analysis. Futur. Oncol..

[6] Gambardella V., Fleitas T., Tarazona N., Cejalvo J., Gimeno-Valiente F., Martinez-Ciarpaglini C., Huerta M., Roselló S., Castillo J., Roda D. (2019). Towards precision oncology for HER2 blockade in gastroesophageal adenocarcinoma. Ann. Oncol..

[7] Tarazona N., Gambardella V., Huerta M., Roselló S., Cervantes A. (2016). Personalised treatment in gastric cancer: Myth or reality?. Curr. Oncol. Rep..

[8] Sohn B.H., Hwang J.-E., Jang H.-J., Lee H.-S., Oh S.C., Shim J.-J., Lee K.-W., Kim E.H., Yim S.Y., Lee S.H. (2017). Clinical significance of four molecular subtypes of gastric cancer identified by the cancer genome atlas project. Clin. Cancer Res..

[9] Wang H.-B., Liao X.-F., Zhang J. (2017). Clinicopathological factors associated with HER2-positive gastric cancer: A meta-analysis. Medicine (Baltim.).

[10] Gomez-Martin C., Plaza J.C., Pazo-Cid R., Salud A., Pons F., Fonseca P., Leon A., Alsina M., Visa L., Rivera F. (2013). Level of HER2 gene amplification predicts response and overall survival in HER2-positive advanced gastric cancer treated with trastuzumab. J. Clin. Oncol..

[11] Boku N. (2014). HER2-positive gastric cancer. Gastric Cancer.

[12] Machado-Neves R., Vale J., Eloy C., Polónia A. (2020). HER2 genomic heterogeneity is a frequent event in gastroesophageal adenocarcinoma and correlates with tumor morphology. Pathol. Res. Pract..

[13] Harbeck N., Gnant M. (2017). Breast cancer. Lancet.

[14] Van Cutsem E., Sagaert X., Topal B., Haustermans K., Prenen H. (2016). Gastric cancer. Lancet.

[15] Oh D.-Y., Bang Y.-J. (2020). HER2-targeted therapies—A role beyond breast cancer. Nat. Rev. Clin. Oncol..

[16] Bang Y.-J., Van Cutsem E., Feyereislova A., Chung H.C., Shen L., Sawaki A., Lordick F., Ohtsu A., Omuro Y., Satoh T. (2010). Trastuzumab in combination with chemotherapy versus chemotherapy alone for treatment of HER2-positive advanced gastric or gastro-oesophageal junction cancer (ToGA): A phase 3, open-label, randomised controlled trial. Lancet.

[17] Hecht J.R., Bang Y.-J., Qin S.K., Chung H.C., Xu J.M., Park J.O., Jeziorski K., Shparyk Y., Hoff P.M., Sobrero A. (2016). Lapatinib in combination with capecitabine plus oxaliplatin in human epidermal growth factor receptor 2—Positive advanced or metastatic gastric, esophageal, or gastroesophageal adenocarcinoma: TRIO-013/LOGiC—A randomized phase III trial. J. Clin. Oncol..

[18] Satoh T., Xu R.-H., Chung H.C., Sun G.-P., Doi T., Xu J.-M., Tsuji A., Omuro Y., Li J., Wang J.-W. (2014). Lapatinib plus paclitaxel versus paclitaxel alone in the second-line treatment of HER2-amplified advanced gastric cancer in Asian populations: TyTAN—A randomized, phase III study. J. Clin. Oncol..

[19] Thuss-Patience P.C., Shah M.A., Ohtsu A., Van Cutsem E., Ajani J.A., Castro H., Mansoor W., Chung H.C., Bodoky G., Shitara K. (2017). Trastuzumab emtansine versus taxane use for previously treated HER2-positive locally advanced or metastatic gastric or gastro-oesophageal junction adenocarcinoma (GATSBY): An international randomised, open-label, adaptive, phase 2/3 study. Lancet Oncol..

[20] Tabernero J., Hoff P.M., Shen L., Ohtsu A., A Shah M., Cheng K., Song C., Wu H., Eng-Wong J., Kim K. (2018). Pertuzumab plus trastuzumab and chemotherapy for HER2-positive metastatic gastric or gastro-oesophageal junction cancer (JACOB): Final analysis of a double-blind, randomised, placebo-controlled phase 3 study. Lancet Oncol..

[21] Patel T.H., Cecchini M. (2020). Targeted therapies in advanced gastric cancer. Curr. Treat. Options Oncol..

[22] Makiyama A., Sukawa Y., Kashiwada T., Kawada J., Hosokawa A., Horie Y., Tsuji A., Moriwaki T., Tanioka H., Shinozaki K. (2020). Randomized, phase II study of trastuzumab beyond progression in patients with HER2-positive advanced gastric or gastroesophageal junction cancer: WJOG7112G (T-ACT Study). J. Clin. Oncol..

[23] Shitara K., Bang Y.-J., Iwasa S., Sugimoto N., Ryu M.-H., Sakai D., Chung H.-C., Kawakami H., Yabusaki H., Lee J. (2020). Trastuzumab deruxtecan in previously treated HER2-positive gastric cancer. N. Engl. J. Med..

[24] Sicklick J.K., Kato S., Okamura R., Schwaederle M., Hahn M.E., Williams C.B., De P., Krie A., Piccioni D.E., Miller V.A. (2019). Molecular profiling of cancer patients enables personalized combination therapy: The I-PREDICT study. Nat. Med..

[25] Pietrantonio F., Fucà G., Morano F., Gloghini A., Corso S., Aprile G., Perrone F., De Vita F., Tamborini E., Tomasello G. (2017). Biomarkers of primary resistance to trastuzumab in HER2-positive metastatic gastric cancer patients: The AMNESIA case-control study. Clin. Cancer Res..

[26] Meric-Bernstam F., Johnson A.M., Dumbrava E.E.I., Raghav K., Balaji K., Bhatt M., Murthy R.K., Rodon J., Piha-Paul S.A. (2019). Advances in HER2-targeted therapy: Novel agents and opportunities beyond breast and gastric cancer. Clin. Cancer Res..

[27] Kim C., Lee C.-K., Chon H.J., Kim J.H., Park H.S., Heo S.J., Kim H.J., Kim T.S., Kwon W.S., Chung H.C. (2017). PTEN loss and level of HER2 amplification is associated with trastuzumab resistance and prognosis in HER2-positive gastric cancer. Oncotarget.

[28] Saeki H., Oki E., Kashiwada T., Arigami T., Makiyama A., Iwatsuki M., Narita Y., Satake H., Matsuda Y., Sonoda H. (2018). Re-evaluation of HER2 status in patients with HER2-positive advanced or recurrent gastric cancer refractory to trastuzumab (KSCC1604). Eur. J. Cancer.

[29] Palle J., Rochand A., Pernot S., Gallois C., Taïeb J., Zaanan A. (2020). Human epidermal growth factor receptor 2 (HER2) in advanced gastric cancer: Current knowledge and future perspectives. Drugs.

[30] Gambardella V., Fleitas T., Tarazona N., Papaccio F., Huerta M., Roselló S., Gimeno-Valiente F., Roda D., Cervantes A. (2020). Precision medicine to treat advanced gastroesophageal adenocarcinoma: A work in progress. J. Clin. Med..

[31] Gall V.A., Philips A.V., Qiao N., Clise-Dwyer K., Perakis A.A., Zhang M., Clifton G.T., Sukhumalchandra P., Ma Q., Reddy S.M. (2017). Trastuzumab increases HER2 uptake and cross-presentation by dendritic cells. Cancer Res..

[32] Janjigian Y.Y., Maron S.B., Chatila W.K., Millang B., Chavan S.S., Alterman C., Chou J.F., Segal M.F., Simmons M.Z., Momtaz P. (2020). First-line pembrolizumab and trastuzumab in HER2-positive oesophageal, gastric, or gastro-oesophageal junction cancer: An open-label, single-arm, phase 2 trial. Lancet Oncol..

[33] Nagai Y., Oitate M., Shiozawa H., Ando O. (2019). Comprehensive preclinical pharmacokinetic evaluations of trastuzumab deruxtecan (DS-8201a), a HER2-targeting antibody-drug conjugate, in cynomolgus monkeys. Xenobiotica.

[34] Mohamed M.M., Sloane B.F. (2006). Cysteine cathepsins: Multifunctional enzymes in cancer. Nat. Rev. Cancer.

[35] Grabsch H., Sivakumar S., Gray S., Gabbert H.E., Müller W. (2010). HER2 expression in gastric cancer: Rare, heterogeneous and of no prognostic value—Conclusions from 924 cases of two independent series. Anal. Cell. Pathol..

[36] Shitara K., Iwata H., Takahashi S., Tamura K., Park H., Modi S., Tsurutani J., Kadowaki S., Yamaguchi K., Iwasa S. (2019). Trastuzumab deruxtecan (DS-8201a) in patients with advanced HER2-positive gastric cancer: A dose-expansion, phase 1 study. Lancet Oncol..

[37] Yamaguchi K., Bang Y.-J., Iwasa S., Sugimoto N., Ryu M.-H., Sakai D., Chung H., Kawakami H., Yabusaki H., Lee J. (2020). 1422MO trastuzumab deruxtecan (T-DXd; DS-8201) in patients with HER2-low, advanced gastric or gastroesophageal junction (GEJ) adenocarcinoma: Results of the exploratory cohorts in the phase II, multicenter, open-label DESTINY-Gastric01 study. Ann. Oncol..

[38] Kreutzfeldt J., Rozeboom B., Dey N., De P. (2020). The trastuzumab era: Current and upcoming targeted HER2+ breast cancer therapies. Am. J. Cancer Res..

[39] Catenacci D.V., Rosales M., Chung H.C., Yoon H.H., Shen L., Moehler M., Kang Y.-K. (2021). MAHOGANY: Margetuximab combination in HER2+ unresectable/metastatic gastric/gastroesophageal junction adenocarcinoma. Futur. Oncol..

[40] Catenacci D.V., Kang Y.K., Park H., Uronis H.E., Lee K.W., Ng M.C., Enzinger P.C., Park S.H., Gold P.J., Lacy J. (2020). Articles Margetuximab plus pembrolizumab in patients with previously treated, HER2-positive gastro-oesophageal phase 1b-2 trial. Lancet Oncol..

[41] Pernas S., Tolaney S.M. (2019). HER2-positive breast cancer: New therapeutic frontiers and overcoming resistance. Ther. Adv. Med Oncol..

[42] (2019). ZW25 effective in HER2-positive cancers. Cancer Discov..

[43] Meric-Bernstam F., Beeram M., Mayordomo J.I., Hanna D.L., Ajani J.A., Murphy M.A.B., Murthy R.K., Piha-Paul S.A., Bauer T.M., Bendell J.C. (2018). Single agent activity of ZW25, a HER2-targeted bispecific antibody, in heavily pretreated HER2-expressing cancers. J. Clin. Oncol..

[44] Lee A. (2020). Tucatinib: First approval. Drugs.

[45] Duchnowska R., Loibl S., Jassem J. (2018). Tyrosine kinase inhibitors for brain metastases in HER2-positive breast cancer. Cancer Treat. Rev..

[46] Bitar L., Zouein J., Haddad F.G., Eid R., Kourie H.R. (2021). HER2 in metastatic colorectal cancer: A new to target to remember. Biomarkers Med..

[47] De Santis R. (2020). Anti-ErbB2 immunotherapeutics: Struggling to make better antibodies for cancer therapy. mAbs.

[48] Waltzman R.J., Sarkar A., Williams E.T., Iberg A.T., Higgins J.T., Willert E.K. (2020). MT-5111: A novel HER2 targeting engineered toxin body in clinical development. J. Clin. Oncol..

